# Construction, comparison and evolution of networks in life sciences and other disciplines

**DOI:** 10.1098/rsif.2019.0610

**Published:** 2020-05-06

**Authors:** Deisy Morselli Gysi, Katja Nowick

**Affiliations:** 1 Department of Computer Science, Interdisciplinary Center of Bioinformatics, University of Leipzig, 04109 Leipzig, Germany; 2 Swarm Intelligence and Complex Systems Group, Faculty of Mathematics and Computer Science, University of Leipzig, 04109 Leipzig, Germany; 3 Center for Complex Networks Research, Northeastern University, 177 Huntington Avenue, Boston, MA 02115, USA; 4 Human Biology Group, Institute for Biology, Faculty of Biology, Chemistry, Pharmacy, Freie Universität Berlin, Königin-Luise-Straβe 1-3, 14195 Berlin, Germany

**Keywords:** networks, construction of networks, network evolution, network comparison, networks in life sciences

## Abstract

Network approaches have become pervasive in many research fields. They allow for a more comprehensive understanding of complex relationships between entities as well as their group-level properties and dynamics. Many networks change over time, be it within seconds or millions of years, depending on the nature of the network. Our focus will be on comparative network analyses in life sciences, where deciphering temporal network changes is a core interest of molecular, ecological, neuropsychological and evolutionary biologists. Further, we will take a journey through different disciplines, such as social sciences, finance and computational gastronomy, to present commonalities and differences in how networks change and can be analysed. Finally, we envision how borrowing ideas from these disciplines could enrich the future of life science research.

## 1. What are networks?

Network science is broadly employed in many fields: from understanding *how friends bond in a party* to *how animals interact*; from *how superheroes appear in the same comic books* to *how genes can be related to a specific biological process*. Network analysis is especially beneficial for understanding complex systems, independent of the research field. Examples of complex biological or medical systems include gene regulatory, ecological and neuropsychology networks. Social networks can include collaborations between scientists or actors, sexual partnerships, or relationships between historic persons, among others. Computational gastronomy is also employing network tools, for instance, in recipe building for a better flavour combination. In finance, the interest of the network analysis often lies in forecasting economic crises or opportunities. Epidemiology often applies network science to investigate how diseases spread, how to avoid pandemics and epidemics [1–3] and for finding the patient ‘zero’ [4–6]. Network dynamics has also been employed to understand how chaos can spread in a system, during a hurricane, for example [7].

Most networks are not static but can change at different timescales. Those changes can happen in a short amount of time: (i) within seconds or minutes, for instance, when the cell reacts to an environmental change or the stock market to the introduction of a new company asset; or (ii) within years, such as recipes or phenotypic traits over the course of a lifetime or evolution. Network changes over a time course can be studied as a dynamic system, a very new approach [8] used, for instance, in social networks to detect community formation [9]. Other research areas have employed comparative and modelling methods to investigate temporal changes in networks.

A related aspect of interest is how particular properties of networks evolve, such as modularity, hierarchy or structures like the hourglass shape [10–13]. There are many different measures and characteristics of networks that can be used to study evolvability. In this review, we will not focus on the evolution of such properties *per se*, but rather on how networks change as a system.

We will start by introducing the basic network terminologies and then explore how biological networks evolve. We will then present some classical and some new studies on network changes in other scientific fields with the intention of comparing across fields how networks change over time and how that is measured. We aim to make suggestions for how approaches from other disciplines could support and improve biological network analyses.

### 1.1. Terminology

While the nature of each system, i.e. what its entities are and what kind of interactions they have, is different, there are common notations. A short dictionary of common network terms can be found in box 1 (marked in bold throughout the text). A brief description of biological terms can be found in box 2 (marked in italics throughout the text).

Box 1. **A short dictionary of network terms.**

A **network** is a pair *G* = (*N*, *L*) of a set *N* of **nodes** connected by a set *L* of **links**.

Two nodes are **neighbours** if they are connected.

The **degree** of a node is the number of nodes it interacts with (the neighbours) [14].

The **weight** is a measure of how strong a particular interaction is [14].

The **direction** of a link specifies the source (starting point) and a target (endpoint) where the interaction occurs [15].

The **strength** of a node is the sum of the weights attached to links belonging to a node [16].

**Hubs** are nodes with a much larger degree compared to the average degree value [16].

A set of highly interconnected nodes is a **module** or **cluster** [17].

Two nodes are connected in a network, if a sequence of adjacent nodes, a **path**, connects them [18].

The **shortest path** length is the number of links along the shortest path connecting two nodes [18].

The **average path** length is the average of the shortest paths between all pairs of nodes [18].

The **diameter** is the maximum distance between two nodes [14].

The **modularity index** is a measure of the strength of the network division into modules when this measure is maximized; it can be used for identifying nodes communities [19].

**Preferential attachment** is the tendency of nodes to form new links preferentially to nodes with a high number of links [20,21].

The probability that a random node in the network has a particular degree is given by the **degree distribution** [18].

A **bipartide graph** is a network in which the nodes can be divided into two disjoint sets of nodes such that links connect nodes from the two sets to each other, but never inside the same set [22]. In those networks, most of the network measures are calculated differently than in a unipartide network.

The **clustering coefficient** describes the degree with which a node is connected to all its neighbours [18].

The **global clustering** coefficient measures the total number of triangles in a network [22].

The **average clustering** coefficient is the average of the clustering coefficient of all nodes in a network [18].

**Centrality** is a set of measures that have been proposed to help to define the most central nodes. It has many interpretations for autonomy, control, risk, exposure, influence and power [23].

**Closeness** centrality is defined as the average distance from a single vertex to all other vertices [24].

**Betweenness** centrality is defined as the total number of shortest paths between pairs of nodes that pass through a particular node [24].

The **topological overlap (TO)** is a measure of how interconnected two nodes are based on common neighbours [17,25], details are given in the Gene (Regulatory) Networks section.

**Global measures** are measures that describe the whole network, for example, degree distribution; average clustering coefficient; path length; modularity index.

**Local measures** are characteristics of individual nodes of a network, such as their degree and centrality.

The **global reaching centrality** (GRC) is defined as the average difference between the maximum local reaching centrality and the local reaching centrality [26].

**Flow hierarchy** measures the heterogeneity of the flow information in a network [26].

Box 2. **A brief dictionary of biology terms.**

**DNA** is the hereditary material of most organisms; usually all cells of an organism have the same DNA [27].

**Genes** are the basic physical and functional units of heredity. They are parts of the DNA and contain the information for producing functional RNAs and proteins. [27].

**Orthologous genes** are genes in different species that originated by speciation events and represent evolutionary equivalents of each other [28–31].

**Paralogous genes** are genes in the same species that originated by gene duplication [28–31].

**Horizontal gene transfer** describes the transfer of genetic material between organisms rather than from parents to offspring. It is most commonly observed in prokaryotes and represents a main factor for species evolution [32].

The **RNA** is synthesized from the DNA but has different properties and functions than the DNA. Some RNAs carry out biological functions in a cell, while others, **messenger RNA (mRNA),** are turned into proteins that fulfil biological functions [27]**.**

After the mRNA is produced, it undergoes substantial changes, such as RNA splicing and 5′ cap addition, leading to a **mature mRNA**, or **secondary mRNA**, which is exported into the cytoplasm for protein synthesis [27].

A **non-coding RNA (ncRNA**) is an RNA that does not encode a protein. ncRNAs often play a role in gene regulation [33].

**microRNAs (miRNA)** are examples of ncRNA; they are involved in post-transcriptional regulation of protein expression [34].

**Proteins** are large, complex molecules that play many critical roles in the body. Proteins are responsible for most of the work in cells and are necessary for structure, function and regulation of the cells. They can act as enzymes, antibodies, transporters, transcription factors, etc. [27].

**Gene expression** is, in short, the coupled process of transcription (from DNA to RNA) and translation (from RNA to proteins) to transform the stored information inside the DNA into proteins [27].

**RNA-Seq** is a technique used to sequence the RNAs in a sample. The result is the snapshot abundance of all RNAs expressed in the sample at a particular time, often called the **transcriptome** [35].

**Sample** is a term that is used very context dependently in biology. Here, a sample is a representative part of an organism, e.g. a piece of tissue or a cell.

**Microarrays,** or **gene chips,** are chips with thousands of tiny spots containing a known DNA sequence. It is used to measure the abundance of mRNAs by eminence of fluorescence [27].

**Transcription factors** are DNA-binding proteins that activate or repress the transcription of particular target genes [36].

**Gene regulatory factors** are responsible for controlling the expression of genomic information and include transcription factors, cofactors, epigenetic modifiers, miRNAs and others [37].

**Second messengers** are molecules that transmit a signal from a receptor protein on the cell surface to target molecules inside the cell. They are parts of signalling cascades, in which usually one second messenger activates several target molecules, thus amplifies the signal [38].

**Systems biology** examines the structures and dynamics of cellular and organismal function, instead of isolated characteristics of a cell or organism [39].

**Operational taxonomic unit (OTU)** is used to classify groups of closely related individuals based on sequence similarity (often 16 s or 18 s ribosomal RNA similarity of microorganisms) [40] and is a helpful concept if species cannot easily be determined.

**Drug repositioning** (or drug repurposing) is the process of redeveloping a compound for use in a different disease [41].

**Yeast-two-hybrid (Y2H) systems** are used to measure protein–protein interaction. Two proteins to be tested for interaction are expressed in yeast; one protein is fused to a DNA-binding domain from a transcription factor while another protein (Y) is fused to a transcription activation domain. If *X* and *Y* interact, there will be a formation of a colony on media used as evidence of the interaction of *X* and *Y* [42].

**Protein complex immunoprecipitation** is an alternative method for measuring protein interactions. It involves immunoprecipitation of the protein bait, purification of the complex and the identification of the interacting partners [43].

**High-throughput mass spectrometry** has the ability to detect a characteristic mass to charge ratio of different substances in a sample. It is used to identify the proteins present in a sample [44,45].

**Chromatin immunoprecipitation followed by sequencing (ChiRP-Seq)** can be used to identify binding sites of transcription factors in the DNA or of histone modification in a genome-wide manner [46].

**Chromatin isolation by RNA purification followed by sequencing (ChIRP-seq)** maps lncRNA interactions to the chromatin [46].

**Genome-wide association studies (GWAS)** are studies where millions of SNPs are tested for association with a particular phenotype using hundreds or thousands of individuals. Those studies shed light on the genetic basis of complex traits [47–49].

**Phenome-wide association studies (PheWAS)** are similar to GWAS; however, the association between a number of common genetic variations and a wide variety and large number of phenotypes are systematically characterized, allowing, for example, to study pleiotropy [50] and improve drug repurposing methods [51].

**Omics** is a term that refers to the study of different areas in biology, and indicates the totality of some kind, e.g. **genome, transcriptome, proteome** … [52].

**Connectome** is a comprehensive map of neural connections in the brain [53].

**Genome** is the complete set of genetic material (including all genes and their regulatory units) present in an organism [54].

**Transcriptome** is the complete set of transcripts present in a sample. Transcripts are RNAs made from active genes, e.g. mRNAs that are turned into proteins. The transcriptome is the major determinant of a cellular phenotype [54].

**Proteome** is the complete set of expressed proteins present in a sample [55].

**Metabolome** is the complete set of small-molecules biochemicals found in a sample [43].

**Trophic levels** is the position an organism occupies in a food chain [56].

A **food chain** is a succession of organisms that use nutrition from other organisms [56].

**Taxa** is a group of one or more populations that forms a unit.

The set of interactions among a set of entities is, in general, called a graph or a **network** [22,57]. In graph theory, each entity is called a vertex, while in network notation, it is called a **node** [22]. Accordingly, the connections between two entities are called edges or **links**, respectively [22]. In this review, we will always use the network notation, unless otherwise specified. The total number of nodes in a network is often denoted as *N* and the number of links in a network is denoted as *L*. While nodes can receive a label, links in general, are not labelled [22] (although, in many cases, weights can also be perceived as a label).

**Figure 1.**
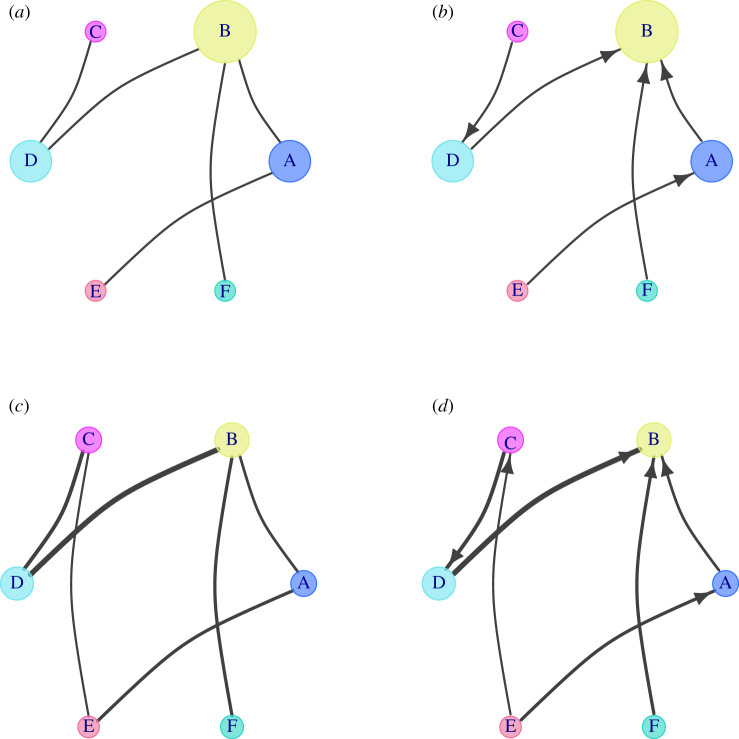
Graph example: (*a*) an undirected, unweighted network and (*b*) a directed, unweighted network. Directionality is visualized by an arrow. (*c*) An undirected, weighted network. Weights are represented by the width of the links. (*d*) A directed, weighted graph. In all panels, the size of the nodes is relative to its degree or strength.

A network can be represented mathematically as an adjacency matrix (usually denoted as *A*), an edge-list or visually as a graph (figure 1). Links of a network can possess a direction (normally depicted by an arrow), which indicates that the interaction is asymmetric, e.g. one gene is regulating another gene, or a person follows somebody else in a social network. Networks with directed links are called **directed** networks, while networks without directed interactions or in which the direction is not known are referred to as undirected networks, e.g. collaboration in the same study or interactions between proteins. The links can also have a **weight** to express the strength of the interaction, which results in a weighted network [22,57]. Usually, the weight is graphically displayed as the thickness or the length of the links.

Networks can also have different dimensions. These dimensions can be understood as layers (or different link types) of the same system [58,59]. For example, in a multi-*omics* multi-layer system, each layer can be constructed using different -omics data (for example, *genomics, transcriptomics, proteomics*, etc.) where the ‘whole’ biological system can be understood as a network of networks [60,61]. The topology and the dynamic properties of the whole network can be changed by simply transforming the weights of the interactions, or by ignoring that nodes can interact in many ways [62–65] also ignoring the node's importance to the system [62,66–69]. The interactions and the dependencies among those layers do not always reflect the real system and are oftentimes more vulnerable to errors than in a single-layer network [70]. However, this extra layer of complexity to network science enables understanding how layers interact and phenomena that cannot be identified using a single-layer system [71,72].

## 2. Comparisons and evolution of networks in life sciences

Recent applications of complex network analysis methods have provided knowledge of the functions and interactions of genes and proteins at the systems level [18,73–75], for example, by analysing protein–protein interaction (PPI) networks [76,77], metabolic networks [78,79] and co-expression networks [80,81]. Moreover, ecological networks investigate how species interact and how they interact with the environment and abiotic factors. All those biological networks are interconnected. Imagine the list of genes and proteins of an organism as a list of ingredients for a meal. Just by listing those ingredients without the complete procedure of how to mix those elements, it is impossible to understand how they should be combined. Similarly, by only having the recipe instructions, without the ingredients, it is not clear what to mix together. In *systems biology*, the concept is similar: a list of genes or proteins provides important information about an organism; however, understanding how those genes and protein interact makes the layers of information much more useful for understanding how organisms develop or react to environmental changes. Systems biology deals with a careful examination of the structures and dynamics of cellular and organismal functions, instead of isolated characteristics of a cell or organism [39]. Biological networks underlie temporal changes—deciphering them are core interests of molecular, evolutionary and ecological biologists.

### 2.1. Protein–protein interaction networks

**Figure 2.**
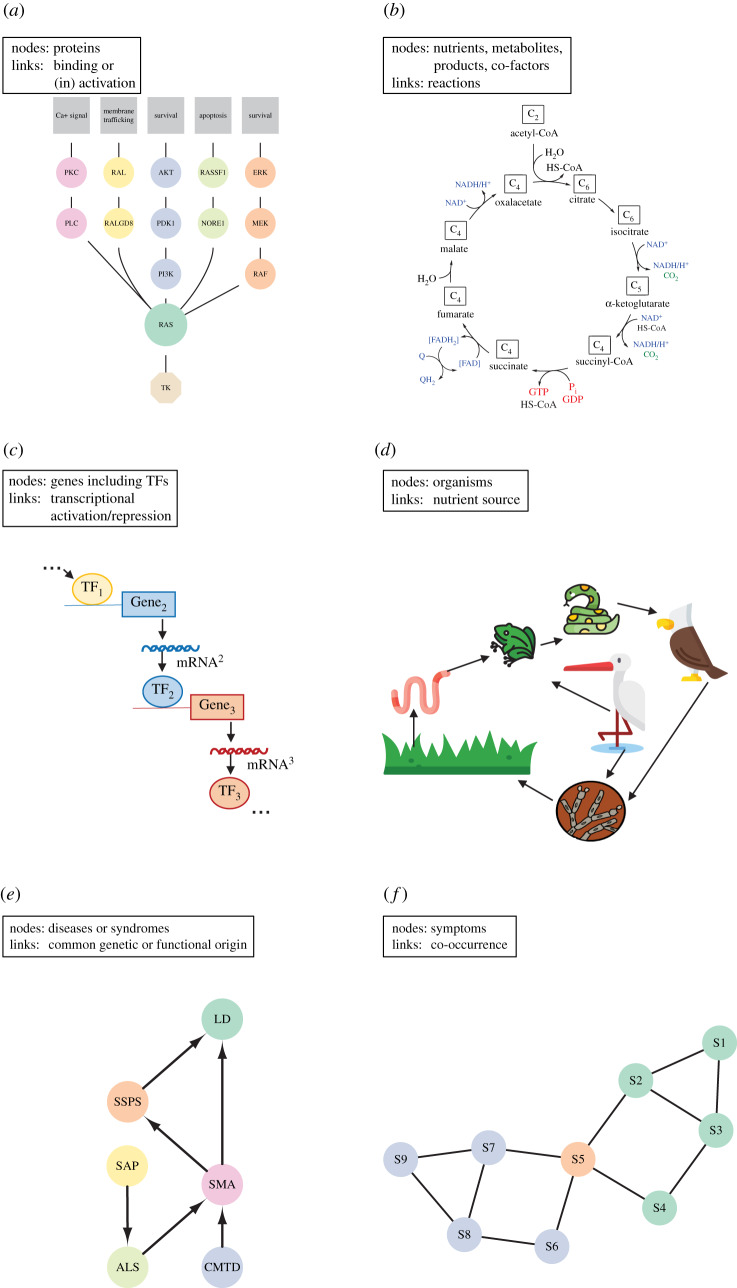
Examples of life science networks. (*a*) Protein–protein interaction networks. Transient interactions occur in signalling pathways. Shown is the RAS signalling pathway, which is initiated by a tyrosine kinase located in the cell membrane. Each protein of the pathway activates the next one and eventually causes particular reactions in the cell; each cascade can lead to a different outcome such as cell survival, apoptosis or membrane trafficking. (*b*) Metabolic networks. They represent a series of biochemical reactions within cells. Shown is the citric acid cycle (adapted from [83]), which is important for gaining energy from food (carbohydrates, fat, proteins), which are metabolized via oxidation of acetyl-CoA into ATP and CO_2_. (*c*) Gene regulatory networks. Shown is an abstract example of a TF that is binding to the promoter of a target gene to turn on its expression. From the genomic information of the target, a messenger RNA (mRNA) is created that gets translated into a protein. If that protein is another TF, it can turn on the expression of another target gene and so on. (*d*) Ecological networks. Shown is a food web with producers (plants), preys, predators and detriments. Nutrients from one organism are used to gain nutrients and energy by the next one in the cycle. (*e*) Disease networks. Shown is the genetic and functional overlap between several diseases. CMTD, Charcot–Marie–Tooth disease; SAP, spastic ataxia paraplegia; ALS, amyotrophic lateral sclerosis; SMA, spinal muscular atrophy; SSPS, silver spastic paraplegia syndrome; LD, lipodystrophy. (*f*) Psychometrics networks. Shown is an abstract example in which symptoms of two disorders (blue and green) mainly cluster by disorder, albeit with a bridge symptom (S5—salmon node) that occurs in both disorders.

In PPI networks, the nodes represent proteins and they are connected by a link if they physically interact with each other [82] (figure 2*a*). Typically, these interactions are measured experimentally, for instance, with the *Yeast-two-hybrid (Y2H) system* [84], or by *protein complex immunoprecipitation* followed by *high-throughput mass spectrometry* [85,86], or inferred computationally based on sequence similarity [87]. PPI can be used to infer gene functions and the association of subnetworks to diseases [77]. In this type of network, a highly connected protein tends to interact with proteins that are less connected, probably to prevent unwanted cross-talk of functional modules [88]. Many methods in network medicine are based on PPI (see below). An example of PPI networks that change in relatively short timescales is signalling cascades in cells (figure 2*a*). Upon a change in a cell's environment, a transmembrane receptor can form a complex with a primary messenger, which leads to the release of a *secondary messenger* that activates further proteins to generate the cellular response. Such PPIs are *sample* dependent, since the set of expressed proteins differs between cells, and information on cell specific expression [89] can be embedded into the PPI [90].

On an evolutionary scale, PPIs can change through gene duplications and sequence divergence. For example, the best characterized eukaryotic PPI network, which was established for the yeast *Saccharomyces cerevisiae* [91,92], showed that the evolutionary older a particular protein is, the more connected over time it is [93]. Interaction networks of some eukaryotic *transcription factors (TF)* became more and more complex due to the duplication of genes encoding for TFs [94]. Results of both studies can be related to the phenomenon known as **preferential attachment**, which states that nodes that already have many links will attract more new links over time than other nodes [20,21]. Duplication of whole *genomes* have also been proposed to be a driving force of gene regulatory network evolution [95,96].

### 2.2. Metabolic networks

To describe metabolic processes, metabolic networks have proved to be valuable. In a metabolic network, the nodes represent the metabolites (biomolecules) and the links a biochemical reaction [97] that is catalysed by proteins (enzymes) (figure 2*b*). These networks contain the stoichiometry of reactions necessary for the synthesis and degradation of basic metabolites or complex compounds such as proteins [98,99].

These metabolic reconstructions have been successfully used in biotechnological applications, mainly targeting the over-production of metabolites [100,101]. The computational approach for analysing this kind of networks is, in general, a constraint-based analysis [102,103]. The usual method for a constraint-based analysis is the flux balance analysis (FBA) that originally was concerned about subsets of the *metabolome* of a cell. FBA models assume that the system is in equilibrium, and can accurately predict the growth rate temporal change [104], interruption in a pathway [105], identify genes that are essential for certain biological functions [106], determine important metabolic pathways [107–109] and aid in synthetic biology studies [110–113]. Another way to construct a metabolic network is by using robust analysis of metabolic pathways (RAMP). Unlike the FBA, it does not assume a steady state for the model and is thus closer to reality. It controls deviations from the steady state by a likelihood function. The implication of this deviation is that it allows computationally study of the functional states of a cell while it converges to a steady state [101]. With the availability of annotated genomes, it became possible to construct genome-scale metabolic networks. They combine inferred or measured gene–protein–reaction relationships, transport reactions and an estimated biomass composition [102,114] and are considered to better comprehend the underlying metabolism of different life forms [104,115].

The structure of metabolic networks can change in response to environmental pressures acting on organisms [116]. **Flow hierarchy**, which is often used in metabolic networks, has also been used to analyse food webs [117] to investigate the evolutionary benefits of the network structure [116]. For example, using simulations in Boolean networks, it has been shown that the cost of keeping a link between two nodes can regulate the hierarchy [118]. This is because maintaining the production of an enzyme that is no longer used comes with certain costs for the individual [119,120]. For the growth of metabolic networks, preferential attachment has also been observed, similar to PPI networks [121].

### 2.3. Gene (regulatory) networks

Biological systems have to regulate when, under which conditions, and how much of a particular protein or RNA is expressed. Interestingly, the molecules that perform this regulation (*gene regulatory factors*) by binding to DNA are themselves encoded in the DNA, thus creating gene regulatory networks (figure 2*c*). Genome-wide binding information can be gained with relatively recently developed methods, such as *ChIP-Seq* or *ChiRP-Seq*, and the effect of the binding can be deduced from analysing expression changes of target genes using RNA-Seq. It is important to note that gene interactions cannot be directly measured, but have to be inferred instead. As an example for network inference, co-expression networks have received much attention [122,123] because they can shed light on the molecular mechanisms that underlie biological processes or on how changes in gene interactions might lead to an altered phenotype, for instance, a disorder.

In co-expression networks, a pair of nodes is typically connected by a link if the genes they represent show a significantly correlated expression pattern across a set of biological samples of interest. They are best built from genome-wide expression data measured by *RNA-Seq* [124] or *microarrays* [125,126]. Often, the links have a weight, which can be calculated from the correlation strength and represent the strength of a gene-pair relationship. The sign of the link can be indicative of whether a gene pair is regulated in the same direction or oppositely controlled [125,126]. Most of the methods for building co-expression networks are based on a similarity measure, such as mutual information or correlation [127–129]. To reduce noise, one can choose to represent the **topological overlap** (**TO**) of nodes instead of each interaction. The TO expresses how similar two nodes are in their set of **neighbours**, such that a link is drawn between two nodes if they share many interactions [17,25]. TO methods, their differences and technical details have been extensively discussed elsewhere [126,130–133]. One needs to be aware that the inference of the links in a co-expression network is a data-driven procedure. Thus, each dataset, even from similar samples, will lead to an independent and different network. Correction approaches are hence needed to reduce noise. Calculating a consensus network from multiple independent networks measured for the same system can serve this purpose [126,134].

Co-expression networks are constantly changing. Biologists are often interested in differences between samples from a healthy and a diseased state or in comprehending changes in gene co-expression during development or evolution. For example, co-expression network analysis has provided insights into neurogenesis [135] and tissue-specific regulatory processes [136–138], into how expression patterns in primate brains have changed over the course of evolution [134,139,140] and how soil treatments can affect rice production [141]. In many studies, theoretical network features, such as the **degree** of a node, degree distribution, **centrality**, **modules** and **hubs** are compared. However, theoretical measures might not have a biological meaning. Instead, biological insight could be gained from the analysis of changes in the topology of the co-expression networks (rewiring), including pinpointing nodes with altered interactions and changed neighbours. Consequently, a method that classifies nodes and links according to the concepts of being present, different or absent in some of the compared networks is beneficial for understanding how different phenotypes are affected by the gene regulatory processes [142]. For a systematic comparison of links or nodes, several methods can be considered: CoDiNA [143], CompNet [144], CoXpress [145], CSD [142], DiffCorr [146,147], Gain [148], MIMO [149], NetAlign [150], SAGA [151] and QNet [152]. While most of these methods allow only pairwise network comparisons [125,142,153,154], CoDiNA and CompNet were developed for the comparison of multiple networks. Table 1 gives a brief overview of the main methods for co-expression network comparisons. However, it is difficult to quantitatively evaluate the accuracy of any of these approaches, since a set of gold standard experimentally validated networks does not exist [153]. Evolutionary analyses of co-expression networks are still rare because comparable *transcriptome* datasets from different species are still sparse [155–157]. Nevertheless, evolutionary differences have been described for the co-expression networks of human, chimpanzee and rhesus macaque pre-frontal cortices [158–160] and plants [161].

**Table 1.** Methods for comparing networks: the columns inform about the statistical methodology used; whether the focus of comparison is on nodes or links; types of networks that are recommended to be compared; and availability of the method.

	methods	nodes/links	can be used for	available
CoDiNA	geometrical transformation, normalized scores for links and classification of nodes	links and nodes	protein–protein interaction, co-expression network, co-occurrence network	R package
CompNet	Jaccard similarities from the union, intersections and exclusive links	links	protein–protein interaction, co-expression network	GUI^a^
CoXpress	hierarchical cluster analysis on the expression values	nodes	co-expression network	R package
CSD	score the links to construct a unified differential co-expression network	links	protein–protein interaction, co-expression network, co-occurrence network	in-house software^b^
DiffCorr	Fisher's *z*-test	links	co-expression network	R package
gain	calculates the Jaccard, Simpson, geometric, hypergeometric and cosine indexes and Pearson correlation for links	links	co-expression network	Web-based
MIMO	subgraph matching	nodes	protein–protein interaction, metabolic network	in-house software^b^
NetAlign	identifies conserved structures from topology and sequence similarity	nodes	protein–protein interaction	Web-based
QNet	computes graph similarities from trees for the nodes based on colouring graph theory	nodes	protein–protein interaction	in-house software^b^
SAGA	computes graph similarities for the nodes	nodes	protein–protein interaction, metabolic network	Web-based

^a^GUI: has a graphical user interface.

^b^In-house software: command line program can be downloaded or requested from authors.

### 2.4. Ecological food networks

Network applications in ecology were born in 1942, when the *trophic levels* were defined [162]. They are the basis for the ecological food networks, also called food webs [163]. Nodes in these networks represent species and their links indicate species interactions (figure 2*d*). Simple food webs have well-defined relationships and topology with, for example, larger predators on the top and a more diverse group of smaller food sources at the bottom [164–166]. However, they are only a small subset of all interactions in an ecosystem [167]. Supplementary and complementary information can be incorporated in the ecological network analysis (ENA) to analyse and model the functions, structures and evolution of complex ecosystems [56,168]. ENA differs from other network models in ecology, in that it assumes an energy and nutrient flow that is modelled through networks [167]. This flux is represented as links, that frequently are directed [167]. As for co-expression networks, plenty of ENA methods are available. They have extensively been discussed in the literature [167–171]. Those methods are generally divided into six categories, of which one is focused on the network topology and pathway types (structure) and five are economy-based with different input/output foci: flow (information diversity framework); storage; environ; control and impact. With the current focus on sustainability and environmental preservation, most of the ENA models use economic models features [172,173]. The model to be chosen depends on the researcher's questions and interests. ENA has been used to better define complex food webs [164,174,175], to understand the effects of external factors in those [164,170,176], to identify taxa specificity and subcommunities in a food network [177], and to uncover interferences of the political sector in sustainability [178–182], among others.

A common interest in ecology is to understand differences between systems, for instance, how species interactions might change depending on the environments [183,184] or climate change. In contrast with co-expression networks, ENA is usually concerned with comparing the network topologies [185], such as degree distribution of the networks and the hubs of each network [183,186]. This renders the comparison of those networks a mostly theoretical framework. Many methodologies have been developed to allow such comparisons [171,185,187–189].

### 2.5. Co-occurrence networks

A complementary method for studying species interactions is co-occurrence networks [183,186,190–192]. Those networks are assembled from the species abundance data of metagenomics studies and can also incorporate other biotic and abiotic information [193,194]. In co-occurrence networks, groups of species identified in the same *operational taxonomic unity (OTU)* correspond to the nodes, and the strength of their interactions with other OTUs are represented by the links [195]. The patterns of co-occurrence can help define the identity of OTUs and how they interact, the biogeography of an environment and functional spatial distribution of the species. Moreover, it also provides information on the spatial effects of environmental conditions and trophic and non-trophic interactions [196]. Those networks are generally built with similarity measures-based methods, such as Spearman or Pearson correlations [186,191,195,197–199], dissimilarity measures, for example, Bray–Curtis [200], probabilistic models, for instance, co-occur [201,202] or other methods, such as the wTO [126] and EcoSim [203].

Similar to co-expression networks, researchers often ask questions such as: is a specific set of OTUs interacting more (or less) in different environments? The first attempt to answer such a question was for a microbiota community by calculating the dissimilarity matrix (1 – correlation). Permutation was applied to remove interactions that could be false positives [199]. Subsequently, different clustering methods such as co-occur [201] and a probabilistic model for co-occurrence [204] were employed to describe microbiota community modules. Those modules were later compared across different environments. It is now common to first **cluster** OTUs to define communities and subsequently compare this cluster structure to identify differences in community composition. However, this does not allow deciphering which OTUs are essential for each condition [167,205,206]. This could be solved by using the wTO method [126] for constructing the networks and using CoDiNA [143] afterward to pinpoint particular OTUs with many condition specific links.

Understanding how ecosystems evolve is of central importance for projecting the effects of environmental or climate change. When time-series data are available, alterations of an ecosystem throughout time can be investigated [207–210]. These analyses focus on the distinction of indirect and direct relationships among the different components and the environments. For example, with NEA (network environ analysis), each compartment in a system has an incoming network and an outgoing network that contains the energy/matter flux [207] similar to the previously described **flow hierarchy** in a metabolic network.

### 2.6. Network medicine

In order to uncover links between diseases, syndromes and comorbidities, network medicine uses a multitude of *omics* information often combined into a multi-layer network. Two diseases are considered to be linked if they have a common genetic or functional origin [211,212] (figure 2*e*). Those connections allow us to understand the interactions between the molecular, symptomatic and social networks that are part of network medicine; allowing the intertwined factors that contribute to individual diseases to be quantified [211]. In general, disease-associated genes or their proteins, respectively, are closely connected with each other, e.g. by being in the same pathway or biological module [77,213–217]. Diseases that are highly phenotypically similar tend to have similar sets of genes associated with them. And genes associated with the same disorder often share cellular localization, functions [214] and are highly co-expressed [218]. Therefore, a subnetwork—called disease module—that can define a disease or a phenotype can be uncovered [214,215,217,219–222].

Network medicine studies, for instance, how a disease progresses. A disease can go through different states, which can cause new diseases, including diseases that, at a first glance, might seem to be unrelated, such as colitis and respiratory insufficiency [223]. Better understanding such progression through states can prevent disease complications and the emergence of other diseases [223–225]. A way to define the *Disease–Disease* progression network is by integrating disease–protein relations, biological pathways, clinical history and biomedical literature into a single network and later calculating the shortest path from any two diseases in that integrated network [223]. The dynamics of cancer metastasis was studied under the light of disease progression by considering the sites where metastasis can arise (given a tumour type) as nodes, defining node size as its hazard and calculating the dynamics of the tumours co-occurrence in every time point [226].

Perturbations of disease modules by drugs can be analysed and related to the drug efficacy and side effects [227,228]. Those disease modules can also be used for drug repurposing, i.e. predicting whether an already approved drug could be used for treating other diseases [229]. To that end, multiple methodologies can be used [230–237]. One example is the signature-based approach, which compares the gene expression profiles under disease and drug-induced states for identifying genes (i.e. the signature) with gene expression reversion [230,238]. For this analysis, the CoDiNA approach described above can also be used. For identification of candidate signatures, one can use a combination of two complementary techniques, such as *genome-wide association studies (GWAS)* and *phenome-wide association studies (PheWAS)*. This approach showed that psychiatric drugs can be repositioned to inhibit the TGF-β pathway [51,239,240], an important pathway that regulates multiple biological processes, such as embryonic development, adult stem cell differentiation, immune regulation, wound healing and inflammation [241]. Genome-scale metabolic network analysis was used to identify drug targets that led to cancer-related drug resistance for cancer therapy [242,243]. Moreover, an integrative method including the protein–protein interactome identified correctly drugs that are already prescribed and also repurpose others [244].

### 2.7. Psychometrics and neuropsychiatry

It is common in psychometrics to explain a set of correlated measures, such as correct responses in cognitive and intelligence tests, or joint occurrence of psychological symptoms, as caused by an unobservable (latent) variable, e.g. general intelligence or depression. The imputation of a value for said variable is the central objective of common psychometric techniques [245,246]. However, the causation structure assumed by these models implies that the latent variable is the unique cause of correlations between observations. This assumption might not be reasonable in clinical and psychiatric settings [247] in which symptoms might cause and modify other symptoms. As an example, lack of sleep and fatigue are associated with depression, and both are items in the Beck Depression Inventory II [248], a 21-question multiple choice self-report inventory, widely used to measure the severity of depression. Yet, it is reasonable to assume that sleep problems might cause some fatigue for physiological reasons not necessarily related to the presence of depression. Therefore, network approaches to investigate the interaction of symptoms have been proposed in the literature. Networks are assembled considering each symptom as a node and the association strength as the link weight [249].

One of the first publications that used network theory in psychometrics (figure 2*f*) investigated the comorbidity of major depression and generalized anxiety disorder and the interplay of their symptoms [249,250]. The symptoms information was taken from the Diagnostic Statistical Manual IV (DSM-IV), a comprehensive manual of 439 symptoms, criteria and language for the classification of mental disorders. This resulted in two well-defined clusters, one associated with each disease. Interestingly, the authors also discovered ‘bridge symptoms’ between both diseases, such as sleep problems, restlessness and concentration problems, which give a possible explanation for the comorbidity of both diseases. In addition, the strength of each node provided insights into which symptoms are more dominant to each separate diagnosis. Similar explorations have been performed in networks linking autism and obsessive–compulsive disorder [251] and the structure of post-traumatic stress disorder (PTSD) symptoms [252]. Also, the entire disease structure of the DSM-IV catalogue has been investigated [250]. Two symptoms were connected if they are used as diagnostic criteria for the same disease. This network revealed one large component comprising almost half (47.4%) of the symptoms, a high degree of clustering and high connectivity. These properties give the network a small world nature, predicting the observed pattern of multiple symptoms interacting in multiple disorders and the observed prevalence rate for comorbidity.

Structural and functional connectivity between brain regions, i.e. the *connectome*, has also been investigated using network methods with the goal to understand the brain [253–259]. A variety of methods exists for investigating the relationship of brains regions with diseases (for a complete review on the topic, refer to [260,261]). For instance, the analysis of functional magnetic resonance imaging (fMRI) can be used for characterization and classification of brain structures and disorders such as Alzheimer's disease, schizophrenia, or bipolar disorder [262]. In connectome networks, nodes can represent the brain regions and links the tracts of white matter that connect these brain regions. The links can also indicate correlations in functional activity [258,263]. Connectomes can be compared between two groups of individuals aiming to identify topological biomarkers [264,265], such as for Alzheimer's disease [266], multiple sclerosis [267], schizophrenia [268], stroke [269] and major depression [270]. An fMRI study of 16 Alzheimer's patients found that increased resting state connectivity 1 day after using a psychedelic was predictive of clinical response [271]. Other patients showed increased amygdala response, suggesting potential for therapeutic efficacy in depression treatment [272]. The comparison of connectomes has been done using network-based statistics (NBS), by which the connectivity between a pair of regions is tested using univariate statistics for functional [273] and anatomical [274] disturbances. Machine learning techniques, such as support vector machine (SVM) and multilink analysis (MLA), which comprise a series of ML algorithms with reduction of dimensionality [275] can also be used for defining the differences between connectomes. Topological information can also be included [276], or groups of networks can be compared with each individual topology [277].

## 3. Evolutionary principles across life sciences

Network approaches are ubiquitous in biological and medical research. However, as seen above, the kind of data researchers deal with and the methods they apply vary across disciplines. Despite the differences, it is common to compare network features, such as degree, degree distribution, centrality and modularity [278–280]. However, it would often be interesting to also compare local features to point out the particular nodes and links that have changed, to identify the key differences between biological or medical conditions, cells or species, and potential master regulators of biological states and pathway. To gain more specific insights about the impacts of changed local features, other types of information can be integrated, such as functional classification or the phylogenetic age of nodes.

**Figure 3.**
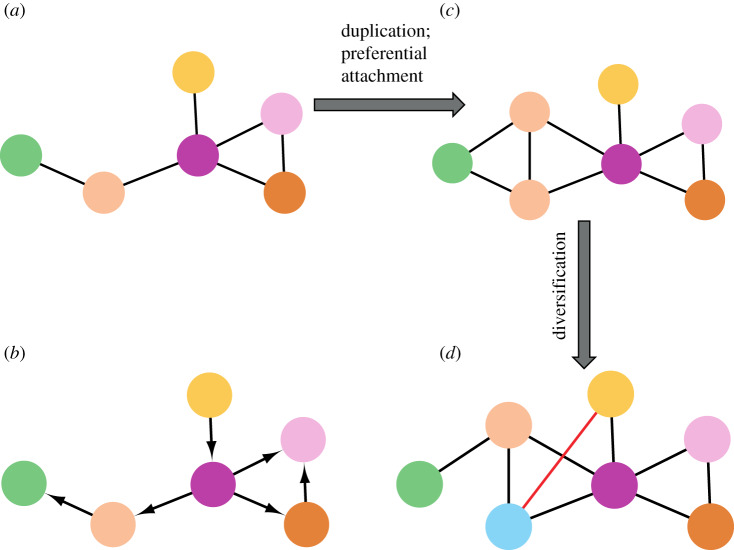
Network evolution. (*a*) An imaginary original network. (*b*) Hierarchical and modular organization. Imagine the network of *a* as a directed network with the yellow node as a key gene initiating a signalling or developmental pathway. Its change or loss would have severe effects on the whole set of functions of the network and hence the organism. Also, a change or loss in the purple node would likely be detrimental, because it regulates most nodes of the network. On the other hand, changes or loss of the green node is expected to cause only little impact on the functions of the network. (*c*) Gene duplication, often following the principle of preferential attachment: a new node (salmon) was created by duplication and attaches to the same neighbours as the original salmon node. It will attach with a higher probability to nodes that already have many links (purple, degree 4, hub in this example). Note: this is not because the genes/proteins with many interactions are more likely to duplicate. The gene duplication process is random. However, it is more likely that one of a hub's neighbours duplicates (because it has more neighbours) [18]. (*d*) Diversification after gene duplication. The new node needs to acquire new functions (or split functions with the node it was copied from) to stay in the genome. Here, it loses the link with the green node but gains a link with the yellow node. This means that the salmon and blue gene now have different functions (albeit overlapping functions to some extend), such that the selection pressure to keep both nodes is high enough. If this is the case, the gene represented by the blue node will be maintained in the genome.

It has been proposed that the evolvability of an organism is related to the hierarchy of the modularity of its molecular networks [115]. This hierarchy of the modularity allows for a better adaptation of organisms to a new environment, may it be by changing the structure of its PPI, metabolic or gene network [281,282]. In particular, links or nodes in one module could be changed without causing too much negative impact on other modules, giving evolution the chance to ‘tinker’ around [283]. The hierarchical and modular organization also allows making predictions on which nodes are more likely to change during evolution [284]. For instance, nodes with fewer links and lower in the hierarchy would cause less severe structural and function impacts on the whole network and thus be more likely to change (figure 3*a,b*). On the other hand, nodes that are high in the hierarchy and are highly connected would be expected to change much less frequently, because their change might often be detrimental to the organism. Moreover, differences in the modularity seem to be correlated to the phylogenetic divergence of organisms. Often, the addition of peripheral pathways results in a loss of modularity during evolution [13].

Evolutionary changes have classically been investigated using the preferential attachment model. Networks with scale-free topology tend to evolve using preferential attachment [15,93]. In molecular networks, this typically means that new nodes are created by gene duplication and are preferentially attached to nodes that already have many links (figure 3*c*). This allows the networks to be more robust during evolution [285], maintaining the same nodes and part of their connections. The topology of a network partly determines how evolvable a network is. A network that is evolvable has the ability to create or allow an adaptive change in a system [286,287], for example, create a potentially phenotypical variation [288]. The particular faith of a new node, i.e. whether it will survive in the genome after duplication, depends on the evolutionary pressures. A new node will originally have exactly the same links as the node it was created from by gene duplication (figure 3*c*). During evolution, most duplicated genes are lost [289]. To escape extinction, nodes need to acquire new functions or split their functions with the original node so that there is enough selective pressure to keep both copies in the genome (figure 3*d*). In this sense, networks do not only grow, but also undergo phases of diversification [10,284].

While scale-free topology can be found in biology [15,18,290–292], gene co-expression networks and some PPI networks are not scale-free [291,293]. These kinds of molecular networks hence require other methods for assessing their evolvability, for which comparative methods or genotype–phenotype networks could be considered. In genotype–phenotype maps [294,295], the nodes represent sequences, e.g. genes or binding sites, and they are linked if they only differ in one position of their sequence. It can then be analysed whether neighbouring sequences encode for the same phenotype, e.g. the same gene function or binding of the same factor. These analyses revealed that such gene networks show a certain level of evolvability and robustness: while changed sequences can still lead to the same phenotype (robustness), it is also possible that evolutionary changes of single sequence positions can create new phenotypes. For these networks, genotype and phenotype information must be available.

Because of the costs and effort involved in generating biological datasets, biologists do not always have enough finely grained data to follow network changes with high temporal resolution. Co-expression networks have sometimes been inferred only for a few time points. With such a low resolution, comparative methods seem to work better than preferential attachment or degree distribution analysis.

Important insights on the evolutionary history of a network can also be gained by using phylogenetic approaches. Here, the evolution of a network created from, let us say, human data, can be traced back by investigating which other species harbour *orthologous genes* to the genes included in the human network. Given a reasonable phylogenetic depth, which is now possible because the genomes of hundreds of species are available, this allows determining at which time in evolution the nodes of the network arose, and in which order they were added to the network. This approach has been applied to, for example, PPI [94] and metabolic networks [116,296–300]. Also, the evolutionary age of links can be inferred using phylogenetic approaches, if comparable networks from multiple species are available. This idea was used when co-expression networks between humans, chimpanzees and rhesus macaques were compared [134]: links present in the human and chimpanzee network were likely also present in the ancestor of humans and chimpanzees about 6 million years ago, while links present in all three species likely existed already in the ancestor of great apes and old world monkeys. Note, that with more species, the loss of nodes or links can also be investigated.

A fundamental mechanism for species evolution is also *horizontal gene transfer*, during which genetic material is exchanged between organisms. Consequently, the evolutionary history of the respective species cannot be accurately displayed in a tree [301]. In such cases, networks have been used to better represent their phylogenetic relationships. A plethora of models for the analysis of such imbricated relationships has been developed [32,302], including identifying differences between gene and species trees and inferring likely sequences of horizontal gene transfer events [303].

## 4. Networks in non-life science and exchange of ideas and approaches

Temporal changes in networks are also investigated in scientific fields other than biology. While we cannot cover all non-biological disciplines in this review, we want to present examples from other disciplines to provide impulses of how ideas developed in one scientific field could be borrowed by other fields to gain new knowledge.

### 4.1. Food-pairing, food-bridging and computational gastronomy

Food-pairing is the concept that ingredients containing similar flavour constitution improve the good taste of a dish [304–306]. Flavour pairing has often been described for the combination of wines with food products [307], but can also be applied to ingredients within the same recipe. Not only the emotion, motivation and craving influence the flavour perception of food [308], it also differs according to culture, climate, geography and genetics. These alterations lead to the development of different cuisines [306,309]. Understanding how compounds inside each ingredient can be paired in a recipe using data science is called *computational gastronomy* [309]. Food-pairing networks can be analysed by treating ingredients as nodes and their co-occurrence in recipes as weighted links [310]. More specifically, the link's weight is given by the number of flavour compounds two nodes (e.g. two ingredients) share in Fenaroli's Flavour Compounds Handbook [311] or The Flavour Bible [312], a chef-curated database. In food-bridging networks, a link is drawn using the shortest path weight of two ingredients. This means that if an ingredient is connected to two other ingredients that do not directly share any flavour compound, they share at least one ingredient that connects both. Another approach is to build **bipartite networks**, where one set of nodes is composed of the ingredients and the other of the flavour compounds. A link then exists if a particular ingredient contains that flavour [305]. Flavour networks are networks that consider flavour compounds shared by culinary ingredients. They are constructed by testing the distribution of recipe sizes, frequency of ingredients, the authenticity of ingredients and if ingredient pairs are different than in a random recipe. The authenticity of a single ingredient is measured by how frequently it occurs in different recipes. Likewise, the authenticity of an ingredient pair is expressed by how frequently that pair appears together in different recipes.

Comparative analyses on the degree distribution of food networks of different cultures revealed that western culture, such as people from North America, Latin America and Southern Europe [305,310], pair their foods according to the food-pairing theory, that is, ingredients with similar flavour go well in the same dish. On the other hand, eastern cultures, such as Indian, Korean and Eastern European prefer to mix different ingredients with different flavours [305,306,310]. Studies are also ongoing for the Arab cuisine [313]. For these cultures, the food-bridging hypothesis applies, which assumes that if two ingredients do not share strong flavour compounds, they can become pleasant in combination through a chain of pairwise affinities [310].

Recipes are also constantly evolving. Similar to many biological studies, e.g. analysing co-expression networks, culinary science has also only few time points that can be compared. Kinouchi *et al*. [314] analysed four cookbooks from different cultures: *Dona Benta* (three editions, from Brazil) [315–317], *Larousse Gastronomique* (France) [318], New Penguin *Cookery Book* (British) [319] and *Pleyn Delit* (medieval) [320]. This allowed them to describe differences among modern recipes, but also to uncover how recipes changed since medieval times and within the modern era, by comparing the three editions of the Brazilian cookbook. Those food-pairing networks were compared based on degree distribution and average recipe size. It is interesting to see that the number of recipes varied from 380 to 1894 recipes and that the average recipe size varied from 6.7 to 10.8 ingredients. In analogy to biological systems, the authors also proposed a copy–mutate algorithm (figure 3*c*,*d*) to model culinary evolution as a branching process. This model considers the time until the process is fixed, e.g. the number of ingredients per recipe, the number of recipes to be mutated, the initial number of recipes in the cuisine and the ratio between the pool of ingredients and pool of recipes. They also included the fitness of each ingredient into their model, which is defined in this context as inherent ingredient properties, such as nutritional value, aspect, flavour, cost and availability. The authors detected statistical patterns in culinary data that are independent of culture and time. For example, in all cookery books, they found that there are ingredients that are not frequently used and could define a hierarchy of ingredients for each culinary. Even though the statistical pattern is quite similar in all the cultures they researched, each one has specific ingredients or recipes that define their uniqueness.

Another study investigated how the late medieval European gastronomy evolved [308]. This is the period before the exploration of the Americas, hence, before the introduction of many ingredients that are common in the European cuisine today, such as potatoes and tomatoes. For the medieval recipes, they grouped recipes from 25 different cookery books from England, France, Germany and Italy from the years 1300 to 1615, from which they manually curated the recipes. They compared the average shared compounds of the medieval recipes with the modern European cuisine and concluded that medieval recipes used food-pairing; interestingly with the pairing having been stronger in that period than nowadays. It is fascinating how the European gastronomy has been evolving in the sense of pairing similar ingredients since the medieval times.

Understanding food composition can also improve health or prevent diseases [321] as shown within a new field of study called *foodome*. For instance, when investigating the effect of a specific polyphenol (EGCG) that is abundant in green tea on type 2 diabetes [322,323] using network measures, it was found that 52 proteins are targets of this polyphenol, out of 83 proteins that are associated with diabetes [321].

### 4.2. Social interaction networks

Many social phenomena can be studied using networks, for example, how scientists collaborate [324,325], how composers write songs [326,327], how people used to get married in the fifteenth century [328], how criminals are associated [329], how friends interact [330,331] and which languages people speak [332]. Compared to biological networks, social networks are in general larger and individuals tend to cluster in communities [333]. The nodes (individuals, groups, organizations) in a social network are linked if they share, for example, values, visions, ideas, contacts, kinship, conflict, financial exchanges, trade, group participation in events, sexual partners, friendships or scientific collaborations [334]. Each one of those social networks can have different properties [335]. In contrast with biological networks, the number of clusters in social networks is often higher than expected by chance [333,336,337]. Links can be measured based on surveys, historical records or otherwise.

In 1929, the Hungarian Frigyes Karinthy hypothesized that only six degrees separate any two people [338]. Later, this hypothesis was tested by asking people to send postcards to acquaintances, which revealed an average degree of separation in a range of 4.4 and 5.7 [339]. In the classical paper [340], three examples are discussed with an average of six degrees of separation. In another classic example, representing one of the biggest Milgram's experiment, a social network was constructed using information collected by Facebook. The Facebook network had, at the time of the experiment, around 721 million active users and 69 billion friendship links [341]. They found that on average the degree of separation of any two people in Facebook is 3.74. To set this in perspective, in a food web, the average degree of separation of any two species is 2 [342], indicating that species are more highly connected to each other creating a higher level of dependency than people.

Changes in popularity among teenagers over time were studied with subjects from the 6th to the 12th grade [343]. Each year, the students' network was created based on a survey asking them to list their best friends. The highest centrality measures can be found in the 6th grade, meaning that an individuals’ number of peers (or popularity) decreases as they get older. Moreover, the older the teenagers get, the more they tend to group in closed clusters that do not share many connections with other clusters.

The movie industry offers many complex systems that can be analysed using network methods. One example is the Hollywood networks. Here, nodes are actors, and they are linked if they appear in the same movie or TV show [344]. Similar to biological networks, the Hollywood network also evolves by adding new nodes via new links to existing nodes [285,335,345]. The Marvel Universe also had its network analysed. Two characters in this network share an interaction if they appeared in the same comic [346]. This network is a fully invented network: characters and their interactions depend mostly on the writer's team and the public critic. Because it does not resemble the real life, it might be expected that this network behaves more closely to a random network than a real-life network. To test this hypothesis, the Marvel Chronology database that includes a catalogue of all significant characters and their appearances after November 1961 [347] was assessed. The most central character turned out to be Captain America. Remarkably, the maximum degree of separation in this network is 5, which is not much different from real-life networks, in which this number is, as previously discussed, somewhere between 3.74 and 6 [22,339–341].

The prostitution network in Brazil was studied using Web forums to understand how this network evolves [348]. They followed the sexual encounter forum from September 2002 to October 2008. The customers' network grew at a rate of six new customers a day, while the sex-seller increased at a smaller rate, five new prostitutes a day. They suggested that the prostitution *career* length is shorter than the sampling timeframe they followed, mainly because the node degrees reached a saturation towards the end, but the customers stayed in the network, indicating that customers use these services for longer than the career length of the sex-seller. The channel they used to gather the data also scores the sex-sellers as ‘bad’, ‘neutral’ or ‘good’, and they showed that professionals with higher average scores attract more clients over time, while there was no difference between mid or bad sex-sellers. The prostitution network also has a preferential attachment [348,349]. Network studies like this one can help to understand how diseases spread through sexual networks [56,350] or social interactions [351] by incorporating more robust epidemiological models. For a discussion on epidemiological models please refer to [352].

How the scientific community collaborates and evolves has also been investigated using a network approach. In these networks, nodes are scientists and a link exists if two scientists co-authored a paper. Scientists collaborate within their own country or internationally. Within the same network, scientists share ideas, techniques and influences [324]. It has been shown that scientists who collaborate more internationally are cited more frequently [353]. Those international collaborations begin more often face-to-face [354], while the following contact and collaborations can be through the Internet. As many biological networks, collaboration networks also evolve by preferential attachment: scientists that have more citations tend to increase their citations over time [20,335,355–358]. The emerging networks behave similarly to a theoretical preferential attachment system. This phenomenon can also be interpreted as a popularity effect among scientists and this behaviour is independent of the field of research. In sociology, Moody followed the behaviour of scientists from 1975 to 1999 [324]. Over time, a decrease in authors who published only one paper and an increase in authors who published more than 11 papers in a lifetime was observed. Unlike in the original preferential attachment model [20,21], the degree distribution in this kind of network shows preferential attachment mostly in the middle of the distribution. The beginning of the distribution (low degree nodes) contains mainly newcomers to the area, while the end of the distribution (high degree nodes) represents the prestigious scientists [357,359,360]. There was also a decrease in the number of papers with only one author, showing that indeed, collaborating in science becomes the norm. Moreover, the average degree increases (people publish more over time) and the average separation degree of the authors decreases over time [335].

**Figure 4.**
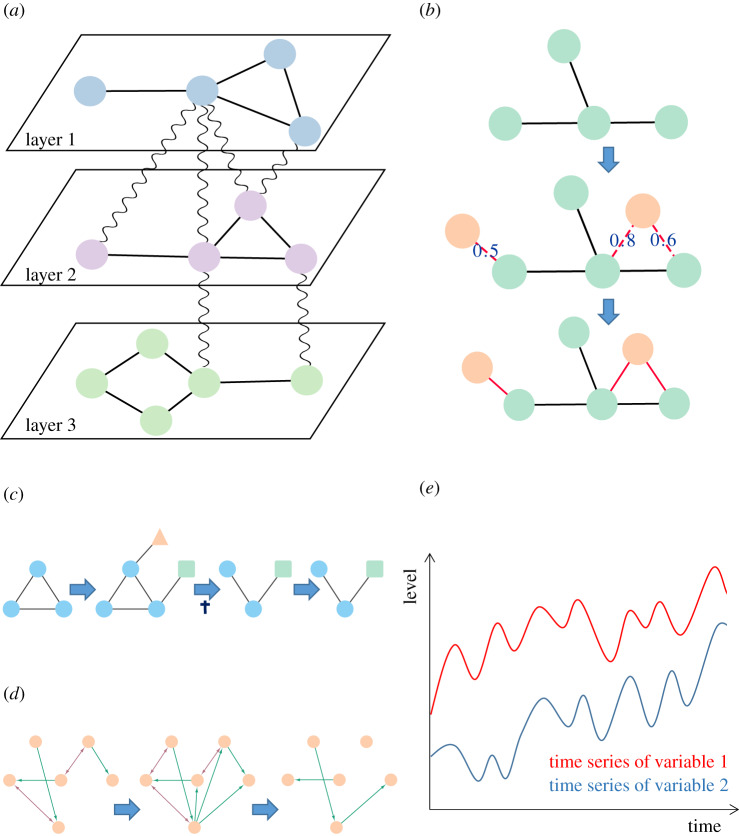
Network analysis methods: depicted are selected methods for network analyses that have been used by different disciplines and can be applied to investigate temporal changes in networks from life sciences data. (*a*) Multilayer networks are used when networks interact or evolve together. Networks can have multiple types of links (the types making up for the layers). A node in one layer can be connected to any node in the same layer (intra-layer links, straight lines) or in any other layer (inter-layer links, curvy lines). Layers could be, e.g. interactions between humans with humans and interactions between humans and bots, or co-expression information and binding site information for TFs (interlayer links would arise when TFs that are co-expressed have binding sites in the same promoter). (*b*) Dynamic network analysis investigates temporal changes in networks that can consist of different types of links and nodes and integrate data from multiple networks. Networks take uncertainties into account by representing the probability that a link exists. Temporal changes are investigated by simulations and modelling approaches. It can be explored with which probabilities new nodes and links are added and hence how likely it is that the network structure changes in a particular way over time. This analysis can be applied to social networks, for instance if it is known, what types of personalities prefer to collaborate. It could also predict probabilities of, e.g. interactions in PPI, if the interactions of a paralogous gene and the sequence differences of the paralogs are known, and considering if the paralog was created by single gene duplication or whole gene duplication. (*c*) Birth and death of nodes considers interactions at a series of time points. Nodes can appear and disappear. Some interactions might be very short-lived and represent no real/strong interactions (e.g. the interaction with the salmon triangle). This can be applied to the analysis of changes in social networks over decades/centuries but also to the evolution of molecular networks, in which genes can be created, e.g. by duplication, and disappear, e.g. by mutations leading to a pseudogene. (*d*) Future developments can be predicted from observing patterns of one-way (green links), two-way (red links), three-way, … , n-way connections, for instance, in financial networks to predict financial crises. The same strategy could be used for species interactions in ecosystems to forecast consequences of environmental changes. (*e*) VAR models (vector autoregressive models) are used for analysing multivariate time series. At least two variables that influence each other and change over time are investigated. Each variable is a linear function of past lags of itself and past lags of the other variables. It has been used, e.g. to estimate economic relationships and to understand the interferences of developing symptoms in psychometrics. It could also be used to discover how subsequent mutations in multiple genes influence each other to, e.g. create cancer or neurodegenerative phenotypes. VAR models could be used in co-occurrence networks in ecology for improved comprehension of how species interact over time.

More information about the complex social behaviour could be gathered by using a multi-layer network approach (figure 4*a*), which integrates multiple networks. For scientific interactions, author–author, author–journal and author–paper interaction networks were combined such way. Analysing publication patterns across the last 100 years, it was revealed that over the last decade, many authors tend to diversify by publishing in more journals [361]. This might indicate that science is becoming more cross-disciplinary and that there is a need to share knowledge across fields. Humans can also interact with bots and vice-versa using social media. This kind of interaction can be either good or bad, depending on how bots are used. A recent example of misuse was detected with twitter data from 22 September to 3 October 2017 [362], the time period when Catalonians voted for or against their independence. A multi-layer approach was used to integrate a human–human interaction network with a human–bot interaction network. It revealed that bots had generated specific content that was mostly negative and targeted the most influential individuals among the group of Independentists in Catalonia [362].

Multi-layer networks start to be used in biology, for instance, by aiming to integrate different types of omics data into one network [363]. For gene regulatory networks, it would be beneficial to more tightly integrate co-expression data with data on TF binding sites, taken from CHIP-Seq data or databases, such as Jasper, Transfac and Encode. CHIP-Seq has been combined with TF expression data to predict the activation status of regulatory elements. This integration is normally studied using a statistical paired expression and chromatin accessibility (PECA) model [364] that can be later experimentally validated [365]. Additionally, non-coding RNA molecules, such as microRNAs, are important regulators of gene expression by binding to DNA, RNA or proteins [366] and their binding site information could be included as well. In this sense, a multi-layer network can be used, where each omics is a layer of the system. Such integrated networks allow for better prediction of diseases [60,61,367], have been used to better understand the regulatory mechanisms in cancer [368], differences in cancer [369] and metabolic perturbation in *Escherichia coli* [370]*.* Below we will envision some ideas on how to enrich the analysis of biological networks by including information from non-biological sciences.

Social networks have also been studied in animal societies, e.g. monkeys [356,371], apes [372,373], zebras [374,375], dolphins [376,377], social insects [378,379] and even in slime moulds [380,381] as well as in other species. For example, ants [382] and bees [383] have specific job assignments inside their communities and change their jobs as they age. In an ant colony, individuals assign themselves to a new job through social interactions with co-workers or through the perception of environmental stimuli [378,382,384–387]. The network interactions in those insect communities are shaped by how the tasks are distributed across individuals [378]. A dynamic network analysis (DNA) (figure 4*b*) approach has been employed to investigate trophallaxis, a common social interaction in highly social insects, during which two individuals orally exchange food [379]. DNA is powerful for investigating temporal social network changes and differs from traditional social network analysis by taking into account different kinds of interactions and levels of uncertainty. Simulations and permutations are used to assess network dynamics. The authors [379] recorded more than 1.2 million social interactions between honeybees to understand the structure and dynamics of information flow in trophallaxis networks and discovered that information flow is faster than expected by chance. While so far mainly used in the context of social networks, DNA should be considered more frequently also for the analysis of molecular networks, for instance, for a better temporal investigation of signal transductions, gene regulation, metabolic networks and co-occurrence networks, which are similarly complex and for which it makes sense to represent probabilities of interactions.

Importantly, social networks can be understood as snapshots at a given time point, and their changes can be studied as differences between those snapshots. Interactions seen only for a short period might be less relevant or even spurious. A crucial aspect to consider is that nodes have a date of birth and death [388] (figure 4*c*). Thus, when the time between the snapshots is long, not all networks would consist of the same nodes, for instance, when there are legal, behavioural and other cultural changes in the society that might reflect in the interactions, making the network comparisons more challenging [388]. Lemercier [388] discusses this problem for cases such as connections between people who live(d) in different time frames, or companies that open or close at different times. Further, the rules can change over time, making connections across nodes easier or not. She suggests that those cases should be studied carefully, and offers three main options to overcome this issue: (i) code for ties and group interactions; (ii) be aware of the boundaries; and (iii) do not differentiate long-lasting attributes from the ones that are steady. The problem of ‘lost nodes’ is also common for biological networks, for instance, when investigating molecular networks that underwent gene deletions, ecological networks with extinct species or in which individuals die, or networks in which a particular gene is not expressed in a certain condition. The suggestions of Lemercier could be applied to the evolutionary analysis of such networks as well.

### 4.3. Finance

Financial markets are deeply and strongly interconnected and hierarchical. From the matching of sellers and buyers over an exchange to the worldwide bank networks, the financial system has all characteristics of a complex network-based system. However, classical economic theory disregarded these properties, relying on agent-based models instead [389,390]. This paradigm was challenged by the subprime credit crisis of 2007–2009. Traditional economic models based on classical economic theory were unable to explain, much less predict, its wide-reaching and systemic consequences, demanding novel approaches to be developed. Network theory stood out as an approach to better monitor and manage financial systems and help anticipate and manage future events [390]. However, this endeavour is easier said than done. While a massive number of transactions occurs daily in every level of the financial system, these data are not readily accessible and rarely sensitive to the information, which led to two main strands of method development. The first strand gathers whatever data are accessible (usually interbank or similar data [391–394]) to model the system as a network and study its properties. The second strand also uses such incomplete data, but tries to infer the missing information to get the full picture of the network and thus derive systemic conclusions [395].

The financial system can be represented as a network with banks being considered as nodes and payments are modelled as directed and weighted links. One study explored the topology of the network of interbank payments done over the United States Fedwire Funds Service prior to the subprime crisis [394]. In this study, data covered the first quarter of 2004, modelling each day as a separate network and resulting in a final combined network of 62 daily networks. This network consisted of 6600 banks and more than 70 000 links. It became apparent that not all nodes are created equally, resulting in an asymmetry with preferential attachment to central banks. The network contained a core of very big and interconnected banks dealing with high-value transactions. Indeed, 75% of all daily values are transferred through an inner network of 66 nodes and 181 links with an even more internal clique composed of just 25 banks to which the remainder of the network connects. If this central clique was disrupted, 78% of all banks would be directly impacted [396]. This illustrates the highly interconnected nature of the financial system and how its connectivity would amplify the impact of the collapse of the mortgage-based securities in the following years. This hierarchical organization is similar to many biological systems, in which a few hub proteins orchestrate many important functions, for instance, RNA polymerases [397], which are important to start transcription of all genes, or the tumour suppressor p53 [398], which prevents mutations in the genome. Mutations in such hubs lead to severe outcomes (as discussed above), for example, individuals that are not viable at all or the development of cancer.

A dream of many scientists is to be able to predict the onset of diseases such as Alzheimer's, Parkinson's or cancer. Potentially, analysis approaches of financial systems might aid with making this dream to become true. By observing the financial system over some time period, it is possible to predict future developments (forecasting) (figure 4*d*). Squartini and colleagues [391] used networks, in which institutions are represented as nodes and payments as links, to study the quarterly intra-bank exposures between Dutch banks between 1998 and 2008, aiming to derive early signs of an upcoming financial crisis. Some signals can be derived by analysing how the number of dyadic (two-way), one-way and absent connections varies over time, especially when correcting for the extreme heterogeneity between bank connections. The authors discovered that there was a marked increase in one-way connections with a notable increase of two- and three-node connection patterns during the pre-2008 crisis period. By contrast, during the crisis, an absence of two-node connections and a decrease in dyadic connections were observed. Another study applied network methods to a bank-firm bipartite credit network from Japanese companies [392]. Credit offers from a financial institution (one set of nodes) to a firm receiving the credit (the second set of nodes) were represented as directed weighted links. Data of a 6-year time period (2000–2005) of around 2600 firms and 200 banks each year were analysed, leading to the conclusion that Japanese firms tend to cluster most of their credit operations into a ‘main bank’, leading to smaller diversification. Similar patterns of clustering were found when analysing the returns from the US publicly traded institutions [399]. In that study, firms are nodes and directed weighted links represent the influence of a given firm into another's liabilities (more specifically, their value at risk). The resulting risk network found three main clusters: a first group of large risk drivers that should be closely monitored by regulatory authorities, a second group of risk takers that provoke little systemic risk but would be hurt by spill over effects, and a third and largest group of companies on the intermediate situation of both risk takers and transmitters that can disseminate and amplify risk into other channels and, as such, should be properly monitored and regulated. The idea of a bank-firm bipartide network proposed in the financial sciences could be transferred to biology by representing gene expression and protein levels as two sets of nodes of a bipartide network. The correlation between a gene's expression and protein levels could be the weight of the links. Using this approach, it is expected that genes and proteins with similar functions and the ones that are disease-related would form separated clusters.

Another powerful approach for forecasting future developments in economy are vector autoregressive (VAR) models [400] (figure 4*e*). They allow capturing the linear interdependencies among multiple time series to study temporal aspects. VAR models can be applied to psychometrics studies, for instance, to investigate how symptoms develop or interfere. The associations between dynamical emotion networks and neuroticism have already been explored using a VAR model [401]. The authors modelled the time series of network data, in which symptoms are nodes and weighted links represent the association between symptoms under the VAR model, producing an overall network for the observed period. This model shows some flexibility, allowing for self-loops representing the feedback loop caused by, for instance, anxiety, in which being anxious might elevate one's anxiety even more. Indeed, results in denser networks for more neurotic individuals revealed a higher occurrence of feedback loops and chain effects on the manifestation of symptoms, leading to distinct emotion dynamics. Applying VAR models to the analysis of molecular networks, they could help with better understanding how subsequent mutations in multiple genes influence each other to, e.g. create cancer or neurodegenerative phenotypes. The same approach could be used in co-occurrence networks in ecology for improved comprehension of how species interact over time.

## 5. Integrating networks across scientific disciplines

In addition to borrowing methods, we also think that the integration of different types of data and networks will allow for completely new biological insights. We already mentioned the potential of integrating co-expression data and TF or non-coding RNA binding site information using multi-layer networks. However, it is still challenging to integrate multiple omics data into one network [363]. With the following additional devised examples, we want to underline that we foresee large potential for multi-layer network approaches (figure 4*a*) to achieve such data integration.

For instance, improvement in understanding how the brain achieves its functionality and of mental disorders could be achieved by combining information from co-expression networks of different brain regions with fMRI and psychometrics networks to better link symptoms to brain regions. Moreover, the symptoms network can also be layered with the co-expression network of patients to better improve diagnostics and treatment. Insights from such multi-layer networks would be valuable for developing better treatments and higher life quality for those who suffer from complex mental disorders. Disease–disease progression networks are integrating networks on disease–protein relations, biological pathways, clinical history, along with biomedical literature for better predicting the future of phenotypes [223]. Being able to predict disease progression would aid precision medicine immensely.

Using a multi-layer approach could also be beneficial to the study of ecological systems, where each layer can represent a different type of interaction among species [402–404]. For instance, understanding how different pathogens, such as viruses or pesticides, interact with their hosts, environment and other organisms could be beneficial for deciphering infection mechanisms [405]. Further, for a more comprehensive view on how insect societies respond to pathogens, ecological networks could be integrated with social interaction networks, molecular information and economic factors. As an example, bees are an important component of ecosystems as pollinators but suffer tremendously from pesticides, virus infection or climate change. It would be instrumental to investigate how such changes in the ecosystem disturb the bee's social network. Further, alterations in the bee's social network could be correlated with molecular network analyses in the same individuals to inform about its health. Together, information from these three types of networks could provide more effective measures for protecting bees.

Finally, to learn more about human natural history, food networks could be combined with social networks of humans, to study how domestication and trading of goods might have affected human societies or the spread of diseases.

## 6. Conclusion

We have reviewed examples of how networks are studied in the life sciences and other fields in a comparative and evolutionary or temporal set-up. In general, a network that is not derived from biological data can often be observed or measured directly and its weights and interactions can be experimentally validated. Hence, noise is a smaller confounding issue and confidence is usually higher in such networks. While some biological interactions can be detected and directly measured, this is not the case for many molecular networks. Hence, they contain a higher level of uncertainty. For example, in a co-expression network, interactions are inferred based on estimating the correlation of gene expression levels. This inference carries more noise than direct measurements in other fields. The reasons for that noise are manifold and include biological differences among individuals, such as gender, age or ethnic group, and technical differences such as sequencing or array platform, data quality and facilities that processed the samples. Therefore, co-expression networks need to be validated by independent studies, and correction approaches should be applied to reduce the noise. Molecular networks are also usually sample specific and require filtering methods [89,90]. These and other reasons probably explain why there are more and other methods for constructing and comparing networks in molecular biology than in other disciplines.

In terms of topology, nodes of psychometrics and financial networks tend to cluster in large components, while social and biological networks have, in general, smaller components. This might mean that the financial market is much more well connected, and its nodes are more highly dependent on each other. The degree of separation is much smaller in species interaction networks than in social networks of people. In psychometrics, many symptoms of mental disorders overlap, and therefore give rise to components that do not distinguish disorders. Financial networks or social networks can benefit from zillions of transactions over several years or from millions of interactions automatically measured every second for a longer time period, respectively.

Currently, a huge drawback for many disciplines for understanding network changes over time is that very few models are using time-series or dynamic network approaches. Most research fields that can draw from a plurality of sequential data, still measure network evolution as if the networks were independent of each other or use a preferential attachment model. This is probably a reflection of the complexity of time-series data and the lack of ready-to-use tools. A way to overcome this paucity would be to build more user-friendly tools that could be more easily applied to unravel network dynamics. Another powerful contribution can be expected to come from the field of data visualization by developing new ways of visualizing complex network data, including the integration of multiple networks and changes over time.

To compare networks more comprehensively, we advocate a stronger usage of multi-layer networks, also including non-biological data. However, to make efficient use of multi-layer networks in biology, it is still necessary to establish better platforms, software and models that can integrate the different layers on the networks or are optimized to include more data. One interesting avenue for dealing with those vast information layers could be to use knowledge graphs [406].

Ideas from evolutionary biology have already inspired at least one network study of the culinary science [314], in which ‘mutations’ of recipes were investigated and ingredients were assigned a ‘fitness’. We argue that, despite the topological and temporal differences between networks, a stronger influx of methods and data from non-life sciences into bio-medicine should advance network analyses in the life science.

## Data accessibility

This article has no additional data.
